# Insomnia and its associations in patients with recurrent glial neoplasms

**DOI:** 10.1186/s40064-016-2578-6

**Published:** 2016-06-21

**Authors:** Matthew E. Robertson, Frances McSherry, James E. Herndon, Katherine B. Peters

**Affiliations:** Departments of Neurology, Portsmouth Regional Hospital, Portsmouth, NH USA; Biostatistics, Duke University Medical Center, Durham, NC USA; Neurology, Duke University Medical Center, PO Box 3624, Durham, NC 27710 USA

**Keywords:** Insomnia, Recurrent, Glial, Neoplasms

## Abstract

**Background:**

Patient with neurological disorders and cancer can develop sleep disturbance, in particular insomnia. Etiology of insomnia is multi-factorial in primary brain tumour patients with possible causes including corticosteroids, psychoactive medications, co-morbid psychiatric/medical conditions, and damage to neuronal tissue.

**Findings:**

To understand better insomnia in recurrent glioma patients, a single-center retrospective analysis was performed looking at recurrent glioma patients from January 2004 to May 2009. Data was extracted and included demographics, clinical factors, psychoactive medications, and co-morbid symptoms. Presence and absence of insomnia complaints was evaluated with other co-morbidities using Chi square and Wilcoxon analyses. Records from 340 recurrent glioma patients were evaluated and 46.8 % (n = 159) indicated presence of insomnia with 20 % (n = 66) actively using medications for sleep. Use of corticosteroids were significantly associated with insomnia (p = 0.0003). Age, gender, tumour location, use of stimulants, antipsychotics, and antidepressants were not significantly associated with insomnia in recurrent glioma patients. There was a trend towards a possible significant association with insomnia to fatigue complaints and use of anti-epileptics, p-values of 0.0501 and 0.0725 respectively.

**Conclusions:**

In conclusion, insomnia is commonly encountered in patients with recurrent glial tumors. Corticosteroid use is associated with insomnia in this population. In light of the frequency of insomnia and its associations, future analysis is warranted into sleep complaints in recurrent glioma patients and its impact on quality of life.

## Findings

### Background

Primary brain tumours represent 1 % of all diagnosed cancers (Ohgaki 2009). In studies in regards to quality of life in patients with primary brain tumours, insomnia is commonly seen (Taphoorn and Bottomley 2005; Taphoorn et al. 2005). This problem not only occurs in high-grade patients but also in patients with low-grade tumours (Gustafsson et al. 2006). In a study by Wellisch and colleagues, they evaluated the incidence of major depressive disorder in patients with primary brain tumours and also found that sleep dysfunction was present in over 50 % of these patients (Wellisch et al. 2002). While sleep dysfunction often accompanies depression, insomnia could be multifactorial in primary brain tumour patients. Chemotherapy, radiotherapy, use of corticosteroids such as dexamethasone, use of antiepileptics (AEDs) and psychoactive medications, and damage to the brain parenchyma either by tumour or surgery could have an impact on sleep architecture. One important question that has been considered is how to treat properly insomnia in patients with primary brain tumours as many of the sleep inducing agents can lead to cognitive difficulty that leads to interference with the quality of life. Therefore, we sought to evaluate complaints of insomnia in recurrent glioma patients and its associations with patient, tumour, medication, and co-morbid symptoms.

### Methods

This was retrospective single-center study to evaluate insomnia and its associations in recurrent glioma patients at the Preston Robert Tisch Brain Tumor Center at Duke from January 1, 2006 to May 1, 2009. This study was reviewed and managed by the Duke Institutional Review Board; this study was deemed to be retrospective and did not require patient consent. The clinical charts of patients that were enrolled in clinical treatment protocols for recurrent glial neoplasms were reviewed. These data reviewed were collected as part of medical care and retrospectively queried. In clinical treatment protocols for recurrent glial tumors, adverse events have been collected using Common Terminology Criteria for Adverse Events (CTCAE), and insomnia was selected as the adverse event to study for this retrospective analysis. Insomnia was captured using the CTCAE version 3 and defined as a disorder characterized by difficulty falling asleep and/or remaining asleep. Severity of insomnia was graded and included mild (occasional difficulty sleeping, not interfering with function), moderate (difficulty sleeping, interfering with function and not interfering with activities of daily living), and severe (frequent difficulty sleeping, interfering with activities of daily living). Patients were defined as having insomnia present or absent. Moreover, subjects in these clinical trials were always queried in regards to the presence of insomnia. Additional data were obtained from medical records and included demographics (age, gender, race, tumour grade, location of tumour), Karnofsky performance score (KPS), number of prior progressions, sleep complaints (such as insomnia, snoring, nightmares, restless legs), sleep disorders (such as obstructive sleep apnea, REM behavioral disorder, restless leg syndrome, narcolepsy), corticosteroid use, AED use, psychoactive medication use, and comorbid symptoms (such as depression, fatigue, and cognitive dysfunction). Of note, KPS is a 0–100 scale used to describe the performance and function of cancer patients in regards to activities of daily living. The rationale for this was to look for associations of these previous pieces of information with the presence and absence of insomnia. Descriptive analysis was performed on all demographic, medication and symptom variables. Insomnia was treated as a binary variable (absent or present) and the following statistical tests were performed: 1. Chi square test for gender, race, tumour grade, side of brain, KPS, number of progressions, corticosteroid use, antidepressant use, stimulant use, sleep aid use, antipsychotic use, AED use, and co-morbid symptoms and 2. Wilcoxon analysis for age. The level of significance was set at p-value <0.05.

### Results

Charts from 340 recurrent glioma patients were reviewed retrospectively. Data is presented in Table 1. In brief, the mean ± standard deviation age was 48.4 years (11.7 years), 225 (66.2 %) were male, and majority (n = 322, 94.7 %) were Caucasian. In terms of tumour grade, 296 (87.1 %) were high-grade gliomas and 44 (12.9 %) were low-grade gliomas. At the time that the data was extracted, a majority of recurrent glioma patients had only one progression (n = 178, 52.4 %) and had KPS of 90–100 (n = 169, 50.6 %). Insomnia was documented as a complaint in the medical record for 159 (46.8 %) recurrent glioma patients. Gender, race, and tumour grade were not associated with the presence of insomnia in this patient population.

**Table 1:** Clinical factors and its associations with insomnia in recurrent glioma patients

Characteristic	Insomnia Absent (N = 181)	Insomnia Present (N = 159)	Total (N = 340)	p-value*
Age				0.6601
N	181	159	340	
Median	50.0	50.0	50.0	
Gender				0.9087
Male	119 (65.7 %)	106 (66.7 %)	225 (66.2 %)	
Female	62 (34.3 %)	53 (33.3 %)	115 (33.8 %)	
Race				0.8122
Caucasian	172 (95.0 %)	150 (94.3 %)	322 (94.7 %)	
Other race	9 (5.0 %)	9 (5.7 %)	18 (5.3 %)	
Histology				0.1483
High-grade glioma	153 (84.5 %)	143 (89.9 %)	296 (87.1 %)	
Low-grade glioma	28 (15.5 %)	16 (10.1 %)	44 (12.9 %)	
Side of brain affected				0.1585
Right	83 (45.9 %)	80 (50.3 %)	163 (47.9 %)	
Left	82 (45.3 %)	73 (45.9 %)	155 (45.6 %)	
Bilateral	16 (8.8 %)	6 (3.8 %)	22 (6.5 %)	
KPS				0.0793
50–80	79 (44.6 %)	86 (54.8 %)	165 (49.4 %)	
90–100	98 (55.4 %)	71 (45.2 %)	169 (50.6 %)	
Number of progressions				0.4868
1	91 (50.3 %)	87 (54.7 %)	178 (52.4 %)	
2	51 (28.2 %)	46 (28.9 %)	97 (28.5 %)	
3+	39 (21.5 %)	26 (16.4 %)	65 (19.1 %)	
Corticosteroid use				0.0003
Yes	59 (32.6 %)	83 (52.2 %)	142 (41.8 %)	
No	122 (67.4 %)	76 (47.8 %)	198 (58.2 %)	
Antidepressant use				>0.999
Yes	53 (29.3 %)	46 (28.9 %)	99 (29.1 %)	
No	128 (70.7 %)	113 (71.1 %)	241 (70.9 %)	
Stimulant use				>0.999
Yes	15 (8.3 %)	14 (8.8 %)	29 (8.5 %)	
No	166 (91.7 %)	145 (91.2 %)	311 (91.5 %)	
Sleep aid use				0.0764
Yes	29 (16.0 %)	38 (23.9 %)	67 (19.7 %)	
No	152 (84.0 %)	121 (76.1 %)	273 (80.3 %)	
Antipsychotic use				>0.999
Yes	3 (1.7 %)	2 (1.3 %)	5 (1.5 %)	
No	178 (98.3 %)	157 (98.7 %)	335 (98.5 %)	
Anti-epileptic use				0.0725
Yes	131 (72.4 %)	129 (81.1 %)	260 (76.5 %)	
No	50 (27.6 %)	30 (18.9 %)	80 (23.5 %)	
Symptom: fatigue				0.0501
Absent	33 (18.2 %)	17 (10.7 %)	50 (14.7 %)	
Present	148 (81.8 %)	142 (89.3 %)	290 (85.3 %)	
Symptom: sedation				0.8895
Absent	131 (72.4 %)	114 (71.7 %)	245 (72.1 %)	
Present	50 (27.6 %)	45 (28.3 %)	95 (27.9 %)	
Symptom: confusion				0.5697
Absent	127 (70.2 %)	116 (73.0 %)	243 (71.5 %)	
Present	54 (29.8 %)	43 (27.0 %)	97 (28.5 %)	
Symptom: mood disturbance				0.2438
Absent	107 (59.1 %)	84 (52.8 %)	191 (56.2 %)	
Present	74 (40.9 %)	75 (47.2 %)	149 (43.8 %)	

There was also no statistically significant observed difference in KPS scale amongst dichotomized insomnia groups (p-value of 0.0793). Ninety-eight (55 %) patients without insomnia had KPS scores of 90-100 while 71 (45 %) patients with insomnia had similar scores. Patients with KPS scores from 50 to 80 ranged from 79 (45 %) in the insomnia absent cohort, to 86 (55 %) in the insomnia present cohort.

Tumour progression is defined radiographically by increased tumor growth on magnetic resonance imaging (MRI). The association between progression of the tumour and the presence or absence of insomnia was investigated. Patients with one progression were similarly represented in the insomnia absent group (91 patients or 50 %) compared with 87 (55 %) patients in the insomnia present cohort. Fifty-one (28 %) patients had 2 tumour progressions without evidence of insomnia, while 46 (29 %) did endorse insomnia. Three or more tumour progressions were documented in 39 (22 %) patients without insomnia and 26 (16 %) patients with insomnia. A relation between an increasing number of tumour progressions and insomnia did not meet statistical significance (p-value 0.4868).

Corticosteroid use was found to significantly impact sleep leading to insomnia. Of the patients on steroid therapy, 59 (33 %) did not have insomnia symptoms while 83 (52 %) had sleep disturbance, reaching a p-value of 0.0003. When further stratified into insomnia severity 47 % (55 patients) endorsed mild insomnia while taking steroids. In patients with moderate insomnia, 57 % (17 patients) were using steroids. Severe insomnia correlated with steroid use in 85 % (11) patients, reaching a p-value of 0.0001.

AED use trended towards statistical significance (p-value of 0.0725) with 131 patients (72 %) without insomnia taking antiepileptic medication, while 129 (81 %) of those with insomnia were taking some form of AED. Grouping insomnia complaints by the number of AEDs taken demonstrated that in patients with insomnia 19 % were prescribed no AED, 59 % were taking one AED and 22 % were prescribed two or more AEDs.

Chi Square analyses were employed to look at several subjective complaints common in the primary brain tumour population in an attempt to correlate presence of these symptoms with insomnia. In patients with insomnia, there was no statistically significant association between documented symptoms of sedation, confusion, and mood disturbance. In regards to the relationship between fatigue and insomnia, there was a trend to significance. Of the 159 patients endorsing some degree of insomnia, 142 (89 %) also complained of fatigue. p-value for this relationship was close to significance at 0.0501.

### Discussion

Sleep disturbance in the form of insomnia remains a major quality of life concern in primary glioma patients. In this retrospective analysis of glioma patients common tumour variables, co-morbid medications, and subjective patient complaints were analyzed as to their roles in causing insomnia. Insomnia is an often minimized, but common symptom in all patients. Insomnia rates amongst healthy adult individuals are 13–33 % (Kaye et al. 1983; Cunnington et al. 2013). In contrast, cancer of any kind has been associated with 30–50 % insomnia rates in newly diagnosed patients, and in 23–44 % of cancer patients 2–5 years after initial diagnosis (Savard and Morin 2001; Savard et al. 2001, 2009). Insomnia in patients with cancer can be precipitated by many factors both from the cancer itself or its treatment (Savard et al. 2015).

Corticosteroid use has been associated with many side effects, including insomnia. Unfortunately, corticosteroid therapy is often a necessary treatment in the treatment of CNS neoplasms. A recent study demonstrated an insomnia rate of 22 % in patients being treated for brain metastases (Sturdza et al. 2008). No study to date has looked at insomnia related to corticosteroid use in a primary brain tumour population. In our current study, there is a clear relationship between corticosteroid use and insomnia. Eighty-three of the 142 total patients taking steroids (52 %) related some degree of insomnia. This represents a higher incidence of insomnia compared to the brain metastasis steroid users, which may relate to differences in amount of corticosteroid given, differing concomitant therapies or different underlying disease processes. There was also a relatively high percentage (47 %) of patients not prescribed corticosteroids who related symptoms of insomnia. This again may relate to disruption of circadian rhythms due to tumour location, burden of psychosocial pressures, or secondary to other therapeutic interventions.

While AED use associated with insomnia trended towards statistical significance in our glioma group, the multitude of pharmacologic options and varying pharmacologic mechanisms make a general analysis of this population difficult. Prior studies have looked at specific AEDs and their effects on sleep in healthy patients and those with underlying epilepsy. Antiepileptic classes such as benzodiazepines and barbiturates have been found to have detrimental effects on sleep (Bazil 2003). Barbiturates can decrease sleep latency and increase sleep continuity (Obermeyer and Benca 1996), but long term use has actually been associated with insomnia and depressed REM sleep (Wolf et al. 1984). Benzodiazepines have been found to decrease slow-wave sleep with prolonged use and withdrawal is associated with insomnia. Phenytoin decreases sleep latency, but has been associated with increased nocturnal awakenings and reduced REM sleep (Legros and Bazil 2003). Interestingly, gabapentin and lamotrigine have both been shown to increase REM sleep (Placidi et al. 2000). Levetiracetam was found to have no effect on number of awakenings, sleep efficiency or amount of REM, but patients subjectively perceive fewer awakenings, more restful sleep and decreased alertness in awakening.

Fatigue is also a common symptom both within the cancer population as a whole, and among patients with primary brain tumours. In a same by Kim et al. a linear regression analysis of sleep disturbance in the form of insomnia was a strong predictor of fatigue as scored on a Brief Fatigue Inventory (Kim et al. 2012). In the primary brain tumour patient cohort studied in this retrospective analysis, insomnia again proved to be a strong predictor of fatigue with over 89 % of patients with insomnia endorsing fatigue.

In conclusion, our retrospective analysis showed that insomnia is a common complaint in recurrent glioma patients, irrespective of grade, and is significantly associated with use of corticosteroids. Discussion of other concomitant medications, such as AEDs, and co-morbid symptoms, such as fatigue, should be performed in patients endorsing insomnia. Evaluation and treatment of insomnia and how it impacts a patient’s quality of life is warranted in this population.

## Abbreviations

AEDs: antiepileptics
CTCAE: common terminology criteria for adverse events
KPS: karnofsky performance status
mg: milligrams
MRI: magnetic resonance imaging
REM: rapid eye movement
